# Uso de concentrados plaquetarios en la regeneración ósea por exodoncia. Revisión narrativa

**DOI:** 10.21142/2523-2754-1101-2023-145

**Published:** 2023-03-26

**Authors:** Magdalena Molina Barahona, Lía Moreno Terreros, Felipe Calle Jara, Cristina Vásquez Palacios

**Affiliations:** 1 Carrera de Odontología, Universidad Católica de Cuenca, Odontología, Cuenca-Ecuador. rocio.molina@ucacue.edu.ec, lmmoreno77@ucacue.edu.ec, fcallej@ucacue.edu.ec, avasquezp@ucacue.edu.ec Universidad Católica de Cuenca Carrera de Odontología Universidad Católica de Cuenca, Odontología Cuenca Ecuador rocio.molina@ucacue.edu.ec lmmoreno77@ucacue.edu.ec fcallej@ucacue.edu.ec avasquezp@ucacue.edu.ec

**Keywords:** extracción dental, regeneración ósea, fibrina rica en plaquetas, plasma rico en plaquetas, dental extraction, bone regeneration, platelet-rich fibrin, platelet-rich plasma

## Abstract

**Introducción:**

La atrofia alveolar producida posexodoncia requiere técnicas de regeneración ósea de calidad, para lo cual es necesaria la aplicación de concentrados plaquetarios, que funcionan como agentes bioactivos en los procesos de preservación alveolar. La utilización de los concentrados plaquetarios demostró ser un osteoinductor óptimo, pues produce tres acontecimientos importantes para el mantenimiento de la estructura ósea.

**Objetivo:**

Analizar el uso de concentrados plaquetarios en la regeneración ósea posexodoncia.

**Materiales y métodos:**

Estudio descriptivo y explicativo. Es una revisión narrativa en la que se recogió información de 26 artículos científicos en base de datos científicos como PubMed, Redalyc, ScienceDirect y Ovid, desde 2012 hasta 2022.

**Conclusiones:**

Los concentrados plaquetarios son materiales fisiológicos que aceleran el tiempo de cicatrización de las heridas posexodónticas. Además, son autólogos, ya que se obtienen del propio paciente, lo cual reduce el riesgo de reacciones posoperatorias, así como la transmisión de enfermedades por vía parenteral, y alivian tanto la inflamación como el edema y los síntomas posoperatorios que se presentan tras una extracción dental. Del mismo modo, ayudan a preservar el reborde alveolar, lo que evita la atrofia a largo plazo.

## Abstract (en)

**Introduction:**

The post-exodontic alveolar atrophy requires quality bone regeneration techniques, and therefore, it is necessary to apply platelet concentrates, which are bioactive agents in the process of preserving the alveolar bone. The use of platelet concentrates proved to be an excellent osteoinducer since it leads to three important effects for maintaining bone structure.

**Objective:**

To analyze the use of platelet concentrates in post-exodontic bone regeneration.

**Materials and methods:**

A descriptive, explanatory study. A narrative review in which data was collected from 26 scientific articles published in scientific databases such as PubMed, Redalyc, ScienceDirect, and Ovid between 2012 and 2022.

**Conclusions:**

Platelet concentrates are physiological materials that speed up the healing time of post-extraction wounds. They are autologous since they are taken from the patient him/herself, reducing the risk of postoperative reactions, and the transmission of diseases by parenteral route. They relieve both inflammation and edema as well as post-surgery symptoms that appear after a dental extraction. They also help to preserve the alveolar ridge, avoiding long-term atrophy.

## INTRODUCCIÓN

En la odontología, es muy habitual realizar un proceso de exodoncia, también conocido como extracción dental, y que se trata de una intervención quirúrgica consistente en el retiro de un diente de la cavidad bucal, el cual, generalmente, representa un problema para la salud estomatológica del paciente. Existen múltiples motivos para llegar a un proceso de exodoncia, pero entre las causas más comunes están las caries profundas, los problemas periodontales, las prótesis, la ortodoncia y los traumatismos dentoalveolares (TDA)^1^.

Cuando se realiza una exodoncia, comúnmente, las paredes óseas tienden a atrofiarse, por lo que pierden las dimensiones en su estructura en sentido vestíbulo-palatino y céfalo-caudal. Esto tiene consecuencias en los dientes adyacentes a la pieza extraída, por la movilidad que sufren las piezas vecinas, lo que dificulta la colocación de implantes por la atrofia alveolar. Además, se presentan problemas al instalar prótesis dentales, entre otros casos. En una revisión sistemática de veinte estudios experimentales, que consistían en observar las variaciones de ancho y alto que sufren los tejidos blandos y duros tras la cirugía, se concluyó que existe una reducción entre un 29 y 63% en el ancho de la cresta alveolar, y un 11 a un 22% en su altura, además de reabsorciones óseas rápidas registradas en los primeros 3 a 6 meses^2^^-^^4^. 

Debido a la atrofia alveolar que se produce tras una exodoncia, para regenerar el hueso alveolar, se promueve la aplicación de concentrados plaquetarios. Los concentrados plaquetarios funcionan como agentes bioactivos que tienen por finalidad lograr la regeneración ósea. Este compuesto contiene numerosos factores de crecimiento, los cuales son liberados lentamente. Según los estudios realizados por Dohan, Doglioli y Sanmmartino, esto se lleva a cabo en un lapso entre 7 y 14 días posteriores a su colación. Además, los concentrados plaquetarios son capaces de reducir el dolor y el edema^2^^,^^5^^,^^6^. 

El objetivo en un proceso postexodoncia es obtener una cicatrización óptima del tejido óseo; sin embargo, podría existir un retraso que se aumentaría el riesgo de contraer infecciones y prolongaría el periodo posoperatorio, lo que impedirá que el paciente regrese a sus actividades cotidianas. Por eso, la aplicación de concentrados plaquetarios ayuda a que el proceso se optimice^7^.

El uso de los concentrados plaquetarios demostró que pueden ser sustitutos óseos óptimos, ya que cumplen varias funciones como favorecer el correcto crecimiento del hueso y preparar un espacio adecuado (también conocido como matriz) para la formación del nuevo tejido óseo. Las propiedades mencionadas promueven la cicatrización de heridas quirúrgicas y hacen que los tejidos perdidos tengan una correcta regeneración^8^.

El plasma rico en plaquetas es un compuesto que proviene del mismo paciente y contiene plaquetas, leucocitos y citoquinas dentro de una fuerte red de fibrina. El proceso para obtener este compuesto consiste en extraer 10 cc de sangre en tubos de ensayo para, posteriormente, separar los componentes señalados, mediante equipos de centrifugado o por tubos de centrífuga estériles^2^^,^^9^. Dohan fue quien introdujo este método en cirugías maxilofaciales como una fuente esencial de compuestos de crecimiento (FC), los cuales mantienen un balance entre los sucesos al momento que los tejidos se reparan, lo que mejora la cicatrización posquirúrgica^10^. El objetivo de esta revisión narrativa fue analizar el uso de concentrados plaquetarios después de un proceso de exodoncia para regenerar el tejido óseo de manera eficaz.

## MATERIALES Y MÉTODOS

### Estrategia PICO

Esta revisión narrativa utilizó el protocolo PRISMA, para lo cual se planteó la pregunta PICO de investigación:

(P): pacientes con exodoncias indicadas

(I): uso de concentrados plaquetarios

(C): la cicatrización biológica del hueso alveolar

(O):la regeneración ósea o preservación alveolar

### Estrategia de búsqueda

**Figura 1:**
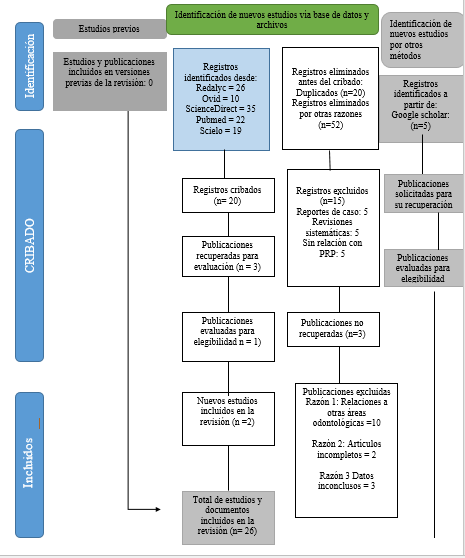
Diagrama de flujo para la obtención de la información

Se realizó una búsqueda de artículos por medio de buscadores digitales como PubMed, Redalyc, SciELO, Ovid, Elsevier y Proquest, desde septiembre de 2012 hasta julio de 2022, y se procedió a su lectura completa, según los criterios de inclusión y exclusión (Figura 1).

### Términos de búsqueda

Los árboles de búsqueda fueron seleccionados para la identificación de artículos; además, se consideró el uso de operadores booleanos: ((("Tooth Extraction [Mesh] OR Extraction, Tooth, Tooth OR Tooth Extractions) AND ("Blood Platelets"[Mesh] OR Blood Platelet OR Platelet, Blood)) AND ("Tooth Socket"[Mesh] OR Socket, Tooth OR Sockets, Tooth OR Tooth Sockets)) AND ("Bone Regeneration"[Mesh] OR Bone Regenerations OR Regeneration, Bone).

### Criterios de inclusión y exclusión

Las publicaciones incluidas fueron aquellas en idioma inglés y español que, en su contenido, abarcaran concentrados plaquetarios empleados en odontología, uso de los distintos tipos concentrados plaquetarios, estudios de casos clínicos con más de 10 pacientes y evidencia científica que evaluara las ventajas en la cicatrización de tejidos mediante la aplicación de concentrados plaquetarios. Se excluyeron los artículos aplicados en animales, pacientes periodontales y en medicina estética.

### Proceso de selección

En una primera etapa, se realizó la búsqueda en PubMed, Ovid y ScienceDirect, con términos Mesh y la antigüedad establecida en los criterios de inclusión. Se identificó 5,000 artículos relacionados con la aplicación de concentrados plaquetarios en estomatología. Posteriormente, se revisaron los resúmenes de los artículos y se descartaron aquellos que no cumplían con los criterios de selección, por lo que se obtuvo un total de 26 artículos científicos para ser analizados a cabalidad.

## DEFINICIÓN DE CONCENTRADOS PLAQUETARIOS

Los concentrados plaquetarios son compuestos naturales que se obtienen mediante procesos de centrifugación de la sangre, generalmente del mismo paciente, lo que da como resultado plaquetas y leucocitos que, esencialmente, segregan factores de crecimiento y citoquinas que activan mecanismos de reparación tisular, gracias a lo cual permiten la cicatrización de una herida y actúan como depósito de células para la regeneración ósea^12^^,^^13^. 

**Tabla 1:** Abreviaturas de los concentrados plaquetarios

Abreviatura	Descripción	Bibliografía
P-PRP	Plasma rico en plaquetas puro (Platelet rich plasma)	Valenzuela J. Efectos de la fibrina rica en plaquetas y leucocitos (l-prf) en distintos procedimientos de cirugía bucal [Tesis de pregrado]. Sevilla: Universidad de Sevilla; 2021. Disponible en: https://idus.us.es/bitstream/handle/11441/134711/TFG%20529-
L-PRP	Plasma rico en plaquetas y leucocitos (Leucocyte and platelet rich plasma)	Valenzuela J. Efectos de la fibrina rica en plaquetas y leucocitos (l-prf) en distintos procedimientos de cirugía bucal [Tesis de pregrado]. Sevilla: Universidad de Sevilla; 2021. Disponible en: https://idus.us.es/bitstream/handle/11441/134711/TFG%20529-
P-PRF	Fibrina rica en plaquetas pura (Pure platelete rich fibrin)	Valenzuela J. Efectos de la fibrina rica en plaquetas y leucocitos (l-prf) en distintos procedimientos de cirugía bucal [Tesis de pregrado]. Sevilla: Universidad de Sevilla; 2021. Disponible en: https://idus.us.es/bitstream/handle/11441/134711/TFG%20529-
L-PRF	Fibrina rica en plaquetas y leucocitos (Leucocyte and platelet rich fibrin)	Valenzuela J. Efectos de la fibrina rica en plaquetas y leucocitos (l-prf) en distintos procedimientos de cirugía bucal [Tesis de pregrado]. Sevilla: Universidad de Sevilla; 2021. Disponible en: https://idus.us.es/bitstream/handle/11441/134711/TFG%20529-
A-PRF	Fibrina rica en plaquetas avanzada (Advanced platelet rich fibrin)	Caruana A, Savina D, Macedo JP, Soares SC. From platele rich plasma to advanced platelet-rich fibrin: biological achievements and clinical advances in modern surgery. Eur J Dent. 2019; 13(2): 280-6.
PRGF	Plasma rico en factores de crecimiento (Concentrated growth factor)	González M, Arteaga-Vizcaíno M, Benito M, Benito M. Aplicación del plasma rico en plaquetas (PRP) y sus derivados en implantología dental y cirugía plástica. Investig Clin. 2012; 53(4): 408-18.

## HISTORIA

La medicina transfusional es una parte de la medicina que se enfoca en el estudio de la transfusión de sangre, lo que da cabida a que se puedan emplear concentrados plaquetarios dentro de los tratamientos para la prevención de hemorragias causadas por enfermedades como la leucemia aguda; asimismo, se usaba en eventos quirúrgicos de larga duración^10^. En 1915, el Dr. Grey utilizó por primera vez la fibrina que proviene de la sangre para controlar la hemorragia producida en la cirugía oral^15^.

A mediados de los años setenta, con el fin de tener una alternativa a las terapias de cicatrización quirúrgica, se comenzaron a emplear los adhesivos de fibrina, que son concentrados fibrinógenos humanos que se catalizaban mediante trombina y calcio. No obstante, el hecho de que estos no provinieran del mismo paciente representó un problema relevante para su aplicación en distintos países; por este motivo, en los años noventa, se solucionó este problema al usar concentrados plaquetarios autólogos, obtenidos por medio de la extracción sanguínea del propio paciente para, posteriormente, aplicar procesos de plasmaféresis y así separar el contenido del fibrinógeno^16^.

En 1974, Ross *et al.* determinaron las plaquetas como potencialmente regenerativas, por lo que fueron los primeros en describirlas como componentes que poseen factores de crecimiento (FC) que, a su vez, tienen propiedades estimulantes para la respuesta mitogenética en el tejido óseo. Aquellos factores son liberados, después de su activación, en el interior de la matriz^15^. 

El plasma rico en plaquetas (PRP) se utiliza desde el año 2000, inclusive anteriormente; sin embargo, en los últimos años, se han realizado continuos estudios experimentales y un gran número de reportes clínicos que han aumentado de forma exponencial su uso. 

El Dr. Choukroun, dedicado a controlar el dolor, se planteó un protocolo de PRF como cicatrizante de heridas, con una compleja reparación y como medio de control del dolor crónico ^15^. En 2006, este mismo científico desarrolló por primera vez la fibrina rica en plaquetas (PRF) con un enfoque en el área de cirugía oral y maxilofacial, sin el uso de anticoagulantes, y obteniéndola directamente de la sangre del paciente luego de centrifugarla. Las plaquetas de la sangre se activan cuando tocan las paredes del tubo y se inicia el proceso de coagulación, una vez que mantiene contacto con el lecho quirúrgico^15^^,^^16^. 

## FACTORES DE CRECIMIENTO

**Tabla 2:** Factores de crecimiento

Factores de crecimiento FC		Bibliografía
EGF Factor de crecimiento epidermoide	Primer factor de crecimiento. Se descubrió en 1960 y puede estimular la proliferación celular mediante cultivo de células. Estimula la multiplicación de células epiteliales al actuar sobre fibroblastos, el tejido liso. Se localiza en la saliva. Se obtiene empleando bioingeniería genética.	González M, Arteaga-Vizcaíno M, Benito M, Benito M. Aplicación del plasma rico en plaquetas (PRP) y sus derivados en implantología dental y cirugía plástica. Investig Clín. 2012; 53(4): 408-18.
TGF-B Factor de crecimiento transformante β	Contiene la molécula TGF β-1. Localizadas en las plaquetas de las células mesenquimales, además en osteoblastos y condrocitos. Incrementan y estimulan a los preosteoblastos y los osteoblastos.	Escalante Otárola W, Castro Núñez G, Geraldo Vaz L, Carlos Kuga M. Fibrina rica en plaquetas (FRP): Una alternativa terapéutica en odontología Rev Estomatol Herediana. 2016; 26(3): 173-8.
PDGFs Factor de crecimiento derivado de las plaquetas	El PDGF está en los gránulos alfa de las plaquetas. Su función esencial es promover la quimiotaxis de monocitos y macrófagos, así como reclutar y proliferar las células mesenquimales y endoteliales.	González M, Arteaga-Vizcaíno M, Benito M, Benito M. Aplicación del plasma rico en plaquetas (PRP) y sus derivados en implantología dental y cirugía plástica. Investig Clín. 2012; 53(4): 408-18.
IGF Factor de crecimiento similar a la insulina	Sintetizadas en el hígado, contienen dos tipos de isoformas: IGF-I e IGF-II. Se acumulan en los gránulos alfa de las plaquetas sanguíneas.	Escalante Otárola W, Castro Núñez G, Geraldo Vaz L, Carlos Kuga M. Fibrina rica en plaquetas (FRP): Una alternativa terapéutica en odontología Rev Estomatol Herediana. 2016; 26(3): 173-8.
VEGF Factor de crecimiento vascular endotelial	Contiene 5 isoformas diferentes, es considerado como el angiogénico más poderoso gracias a su isoforma VEFG-A.	González M, Arteaga-Vizcaíno M, Benito M, Benito M. Aplicación del plasma rico en plaquetas (PRP) y sus derivados en implantología dental y cirugía plástica. Investig Clín. 2012; 53(4): 408-18.
FGF Factor de crecimiento fibroblástico	Presente en la saliva. Contiene dos isoformas: αFGF y βFGF. Induce la epitelización al actuar sobre los fibroblastos y el músculo liso.	Escalante Otárola W, Castro Núñez G, Geraldo Vaz L, Carlos Kuga M. Fibrina rica en plaquetas (FRP): Una alternativa terapéutica en odontología Rev Estomatol Herediana. 2016; 26(3): 173-8.

## CLASIFICACIÓN DE CONCENTRADOS PLAQUETARIOS

Para clasificar los concentrados plaquetarios, según los autores Dohan y Ehrenfest, es necesario considerar los siguientes parámetros: el equipo de centrifugado, el contenido de los concentrados plaquetarios (plaquetas, leucocitos, citoquinas), las redes de fibrina que soportan los PRP y el leucocitario durante su aplicación quirúrgica.

Con base en lo mencionado, se clasifica los concentrados plaquetarios de la siguiente forma^10^: 

### 1. Plasma rico en plaquetas (PRP)

Se considera la primera generación de concentrados plaquetarios. Se prepara tomando entre 20 y 60 ml de sangre del paciente; luego se realizan dos etapas de centrifugación: la primera separa los concentrados plaquetarios de los eritrocitos y la segunda consiste en separar completamente los glóbulos rojos del plasma rico en plaquetas. Para su aplicación tópica, es necesario añadir un agente activador como el cloruro de calcio, que activa la polimerización del coágulo de fibrina antes de aplicar el gel en el área deseada^17^.

### 2. Plasma rico en plaquetas puro (P-PRP)

Fue el primer método que se utilizó para producir concentrados plaquetarios tópicos, con el nombre de plasmaféresis. El procedimiento es intrahospitalario ya que, para la filtración sanguínea, el paciente se conectará a un aparato hasta obtener la cantidad de plaquetas deseada. También se usa una bolsa de sangre que se recolecta con anticoagulantes, la cual pasa a un dispositivo que separa la células mediante centrifugación y tiene un lector que detecta los leucocitos en el suero para recogerlos automáticamente^18^.

### 3. Plasma rico en plaquetas y leucocitos (L-PRP)

Es una fracción autóloga de sangre que contiene plaquetas y concentrados de leucocitos. Ha sido utilizado en el campo de la cirugía por cerca de 20 años debido a sus propiedades curativas, basadas en el aumento de las concentraciones de factores de crecimiento y albúminas. Al utilizar los compuestos activadores como la trombina, los gránulos alfa contenidos en las plaquetas aumentan el número de factores de crecimiento, como PDGF, TGF-β, IGF, EGF y VEGF, los cuales desempeñan un rol importante para la cicatrización de los tejidos óseos^18^^,^^19^. 

Este agregado se realizó con el fin de sustituir los métodos de obtención anteriores y hacer posible el uso de los concentrados plaquetarios sin el apoyo de un laboratorio de transfusión. Para su preparación, es necesario pasar el contenido por dos etapas de centrifugación: la primera separa los glóbulos rojos del plasma puro en plaquetas y en leucocitos, para luego pasar a su segunda etapa de centrifugado^18^.

### 4. Fibrina rica en plaquetas (PRF)

Fue introducida por Choukroun en 2001 y pertenece a la segunda generación de concentrados plaquetarios, obtenida de una membrana de fibrina, y cual hace que esta promueva la angiogénesis y, con ello, la cicatrización del tejido. Se considera que el procedimiento para la preparación del PRF es simple y de bajo costo, por lo que constituye una buena alternativa terapéutica que usa material autólogo, lo cual favorece la cicatrización y regeneración de los tejidos duros y blandos. Este concentrado tiene un efecto antinflamatorio gracias a la presencia de leucocitos.

Según los métodos, preparar este concentrado posee más ventajas sobre el PRP, ya que solo se recogen de 5 a 10 ml de sangre y se centrifugan en vasos estériles^15^^,^^17^.

### 5. Fibrina rica en plaquetas pura (P-PRF)

Es de segunda generación y se obtiene la mucosa de fibrina, capaz de realizar regeneración ósea. La fibrina rica en plaquetas libera FC (factores de crecimiento), lo que ayuda al proceso de regulación de los tejidos y promueve la cicatrización^20^. Para obtener este concentrando se necesita un anticoagulante como el citrato sódico y un gel separador (polímero), colocados en el tubo que contiene la sangre para después centrifugarla y obtener 3 capas que caracterizan a los glóbulos rojos. Después, la capa de leucocitos y PPP se transfiere a un segundo tubo con cloruro de calcio en donde se activará la coagulación^18^.

### 6. Fibrina rica en plaquetas y leucocitos (L-PRF)

Se le considera de segunda generación. El L-PRF se origina sin el uso de anticoagulantes ni gelificantes durante su preparación, lo cual reduce el riesgo de presentar infecciones o reacciones alérgicas a los componentes. Al no usar ningún aditivo, es más resistente en el tiempo y su manipulación es sencilla. Este método permitirá obtener varios coágulos que contienen el L-PRF^18^^,^^20^^,^^21^. 

### 7. Fibrina rica en plaquetas avanzada (A-PRF)

Es un nuevo concentrado introducido por Choukroun en 2014, que se obtiene mediante la centrifugación, pero con la característica de que el régimen de giro es menor (1300 rpm/8 minutos), lo que hace posible el aumento en la concentración de factores de crecimiento. El hecho de reducir las revoluciones y el tiempo en el proceso ocasiona que la membrana sea más ligera y de fácil adaptación celular^22^^,^^23^. 

El objetivo del A-PRF es recopilar glóbulos blancos (linfocitos, neutrófilos), ya que estos distribuyen adecuadamente las plaquetas y también tienen relación con que su vascularización sea mejor y más rápida^22^.

### 8. Plasma rico en factores de crecimiento (PRGF)

En el proceso de hemostasia, las plaquetas de la sangre son las encargadas de liberar factores de crecimiento; cuando han pasado 10 minutos de este evento, estas comienzan a segregar hasta el 95% de dichos agentes durante varios días después de la lesión. Recibe su nombre gracias a la gran cantidad de factores de crecimiento existentes en el plasma^9^. 

Estos concentrados son la generación más reciente introducida en 2005 por Chen y Jiang. Para su preparación, se necesita únicamente una etapa centrifugación que separará la sangre en 3 partes: en la parte superior del tubo queda el PPP (plasma pobre en plaquetas); en el centro, el gel de PRGF; y en el fondo del tubo de ensayo, los eritrocitos. Además, gracias a este nuevo método de centrifugado se permite una mayor liberación de los FC debido a la ruptura de plaquetas durante dicho proceso^17^. 

### Ventajas y desventajas de concentrados plaquetarios

**Tabla 3:** Ventajas y desventajas de concentrados plaquetarios

Ventajas y desventajas de los concentrados plaquetarios		
Ventajas	Desventajas	Bibliografía
Acelera el tiempo para curar las heridas posquirúrgicas.	Para su obtención, se requiere de equipos y material sofisticado.	Cruz Molina C, Castro Rodríguez Y. Resultados de los concentrados plaquetarios en la regeneración ósea guiada. Rev Cuba Investig Biomed. 2020; 39(2): 1-20.
Es autólogo, ya que se obtiene del propio paciente.	Es limitada la cantidad de membranas que se pueden extraer.	
Poseen liberación de factores de crecimiento prolongado extendido (7 días *in vitro*).		
Alivia el edema y dolor post quirúrgico.		

### Obtención del plasma rico en plaquetas

Se define a la centrifugación diferencial, como un proceso de separación celular, el cual se consigue gracias al PRP (plasma rico en plaquetas). El mencionado proceso se realiza extrayendo la sangre del paciente para luego separarla en distintas fases y considerando algunos protocolos. Además, se emplean equipos como los siguientes^9^:

• Separador celular de densidad de gradiente
• Equipo PGRF para la centrifugación y preparación del plasma

Por otra parte, se muestran tres fases del proceso para la obtención de concentrados plaquetarios^9^:

• Localización venosa
• Extracción sanguínea
• Separación celular

Localización venosa y extracción sanguínea. Antes de comenzar el proceso quirúrgico, se extrae sangre del paciente de la zona antecubital de la arteria radial. 

Separación celular. Un profesional de la salud, con la ayuda de equipo especializado, se encarga de llevar a cabo la fase de centrifugación para obtener la máxima concentración por unidad de volumen de plaquetas, con la finalidad de que no se active el contenido de manera prematura. La configuración de la velocidad angular del equipo de centrifugado dependerá del tipo de protocolo de obtención, ya sea de una o dos fases de centrifugado^9^. 

### Obtención de fibrina rica en plaquetas

Para obtener la fibrina rica en plaquetas, se extraen del paciente 10 ml de sangre, la cual se vierte en tubos de ensayo sin aditivos. El número de membranas es directamente proporcional a la cantidad de tubos extraídos. Posteriormente, se realiza la centrifugación durante 10 a 13 minutos, en tres fases, tras los cuales se obtendrán, en la base del tubo, los glóbulos rojos; en la parte central, el PRF, y en la parte superior, las plaquetas^24^. 

Un parámetro muy relevante en este procedimiento es la velocidad con la que se obtiene la muestra de sangre y la centrifugación, ya que, al no usar anticoagulantes, el proceso de coagulación se inicia en cuanto la muestra se vierte en el tubo de vidrio^24^. 

### Colocación de concentrados plaquetarios posexodoncia

Para un proceso de exodoncia con aplicación de concentrados plaquetarios, existen tres etapas fundamentales.

1. Etapa prequirúrgica. Es necesaria una evaluación clínica estomatológica, con el objetivo de analizar, principalmente, la evolución de la densidad ósea del paciente mediante la toma de una radiografía antes y después de realizar el proceso de exodoncia. 

Por otra parte, se realiza el proceso de producción de los concentrados plaquetarios, mediante la extracción y procesamiento de la sangre, de lo cual resulta un gel con los concentrados plaquetarios que se colocará en los alveolos tratados^8^. 

2. Etapa quirúrgica. En esta etapa se practica el proceso de exodoncia (extracción del diente afectado) para, inmediatamente, aplicar el gel de los concentrados plaquetarios con sus diversos protocolos en el alveolo. Finalmente, se sutura el área tratada^8^. 

3. Etapa posquirúrgica. Consiste en realizar un seguimiento clínico y radiológico después de cuatro, ocho y doce semanas de la cirugía, a fin de validar la efectividad del uso de los concentrados plaquetarios aplicados en la zona^8^.

## DISCUSIÓN

En este artículo se analizaron los usos y los tipos de concentrados plaquetarios que se usan para la regeneración ósea posexodoncia, para identificar la eficacia de los compuestos de crecimiento en el mencionado tratamiento. 

De acuerdo con el estado del arte -que sugiere la utilización de la fibrina rica en plaquetas como alternativa de tratamiento de gran efectividad para la regeneración ósea en procesos quirúrgicos como la exodoncia-, se afirma que el PRF es un compuesto fisiológico y autólogo, por lo que no es necesario el uso de anticoagulantes en su contenido; los lechos quirúrgicos cicatrizan en menos tiempo; se presenta una tendencia a la liberación de compuestos de crecimiento (FC) varios días después de la lesión, lo que ayuda al desarrollo de la regeneración de los tejidos; y se alivian el edema y dolor posquirúrgico^10^. La membrana de fibrina permite la protección mecánica de la zona quirúrgica y, biológicamente, crea una interacción con los mecanismos fisiológicos de cicatrización de la herida^26^. 

Por otra parte, debido al alto costo para la obtención, se determinó que la fibrina rica plaquetas (PRF) constituye una mejor alternativa que el plasma rico en plaquetas (PRP), ya que en este último es necesaria su activación mediante el uso de anticoagulantes como el citrato dextrosa; no obstante, se usa con frecuencia en procesos quirúrgicos^18^.

Una diferencia entre la fibrina rica en plaquetas y el plasma rico en plaquetas es que el PRF requiere mayor tiempo para la liberación de compuestos de crecimiento, debido a que la red de fibrina es fácil de manipular; esto hace que su proceso sea más rápido sin necesidad de anticoagulantes, por lo cual es un material más fiable. Este proceso tiene más beneficios ya que, al provenir de una fuente fisiológica, es un gran agente coagulante que se origina de vasos sanguíneos del propio paciente; de esta forma se reduce el riesgo de contraer infecciones^15^^,^^18^^,^^22^. 

Varios estudios han comparado el PRF en distintas etapas para la preservación del reborde alveolar posexodoncia, y se ha demostrado la preservación del ancho de la cresta utilizando fibrina rica en plaquetas en comparación con la cicatrización fisiológica (coágulo de sangre)_^(27)^_ . 

**Tabla 4:** Ventajas y desventajas de cada concentrado

CONCENTRADOS PLAQUETARIOS		
Concentrado	Ventajas	Desventajas
P-PRP	Es autólogo, sin preocupaciones de enfermedades transmisibles	Es un procedimiento intrahospitalario; se tiene que permanecer conectado a una máquina para la extracción.
L-PRP	Presenta alto contenido de factores de crecimiento y albúminas.	Se usa un aditivo como la trombina; no se necesita apoyo de un laboratorio para su obtención.
P-PRF	Muestra alto contenido de fibrina en la membrana.	Se usa citrato trisódico como anticoagulante.
L-PRF	No se usa ningún tipo aditivo; la membrana es más resistente al tiempo y manipulación.	Se obtiene poca cantidad.
A-PRF	El tiempo de obtención es de 8 minutos.	Requiere mayores cuidados en el proceso de centrifugación.
PRGF	Solo se realiza una etapa de centrifugación.	Para su activación, se necesita la aplicación de cloruro cálcico.

Según los procesos de obtención de los concentrados plaquetarios, el plasma rico en plaquetas se obtiene a nivel intrahospitalario, por medio de transfusión de sangre, lo que hace que sea tedioso e incómodo para el paciente, además de tener un alto costo. Por el contrario, el plasma rico en plaquetas y leucocitos se creó para sustituir el método anterior sin la necesidad de un laboratorio de transfusión, sino mediante la extracción de sangre para posteriormente utilizar equipos de centrifugado y así obtener el concentrado plaquetario^18^. Por su parte, la fibrina rica en plaquetas tiene la ventaja de que, gracias a su malla de fibrina, permite una liberación más prolongada de los factores de crecimiento, por lo que su manipulación es fácil. Finalmente, el A-PRF utilizando menos tiempo (8 minutos) y disminuye la cantidad de fuerzas aplicadas sobre el concentrado plaquetario, lo que puede aumentar la cantidad de células contenidas en la matriz^15^^,^^22^.

## CONCLUSIÓN

Los concentrados plaquetarios son compuestos naturales que se obtienen del propio paciente y se pueden utilizar para la cicatrización más acelerada de tejidos después de realizar procesos quirúrgicos, lo que reduce el tiempo de dolor e inflamación postoperatorios. 

Al analizar la aplicación de la fibrina rica en plaquetas, se evidenció, con base en la literatura, que tiene múltiples ventajas en comparación con el plasma rico en plaquetas, tales como el menor tiempo que se emplea en los procesos de obtención y el no usar ningún aditivo para facilitar su manipulación.

Se llegó a la conclusión de que la aplicación de concentrados plaquetarios dentro del área de odontología se emplea en muchas áreas, como la posexodoncia, para recuperar y cicatrizar de forma más acelerada los tejidos afectados durante el procedimiento. Asimismo, se comprobó que se usa durante la aplicación de implantes y en la periodoncia para ayudar a las terapias regenerativas. Por ello, los concentrados han tenido una gran evolución durante estos años, pues se quiere mejorar los procesos para su obtención y hacerlos así más accesibles y fáciles de aplicar. Esto se ha logrado gracias a los estudios experimentales de múltiples investigadores que han descubierto las ventajas de aplicarlos.

## Fuente de financiamiento:

Esta investigación fue autofinanciada
